# Retroperitoneal mesothelial cyst misdiagnosed as a congenital choledochal cyst for an infant patient: A case report and literature review

**DOI:** 10.1016/j.ijscr.2020.04.075

**Published:** 2020-05-08

**Authors:** Min Yang, Bo Xiang, Xiao-long Xie, Ke-wei Li, Fu-yu Li

**Affiliations:** Department of Pediatric Surgery, West China Hospital of Sichuan University, Chengdu, Sichuan Province, People’s Republic of China

**Keywords:** Mesothelial cyst, Congenital choledochal cyst, Misdiagnosis, Da Vinci surgical system, Infant

## Abstract

**Introduction:**

Mesothelial cyst (MC) is very uncommon in clinic, which could occasionally occur in peritoneal, retroperitoneal or even pleural cavity.

**Presentation of case:**

We hereby described an infant patient with retroperitoneal MC who was misdiagnosed as congenital choledochal cyst by both radiologists and clinicians. A choledochal cyst resection with hepatojejunostomy under the da Vinci surgical system was routinely prepared for this patient but a local resection for the whole lesion was unexpectedly performed during the operation. The diagnosis of retroperitoneal MC was confirmed by the pathological analysis from surgical specimens.

**Discussion:**

MC in small size is usually asymptomatic and sometimes detected by imaging examinations which manifests a well-boundary, watery-density and cystic lesion with no enhancement and is often misdiagnosed as lymphangioma or pancreatic pseudocyst. For pediatric patients, MC can occur in the diaphragm and liver in the literature, while retroperitoneal MC around hepatoduodenal ligament for an infant has never been mentioned before. We reported the first case of a retroperitoneal MC around hepatoduodenal ligament for an infant who was preoperatively misdiagnosed as choledochal cyst surgically treated by the da Vinci surgical system.

**Conclusion:**

Retroperitoneal MC around hepatoduodenal ligament should also be considered and resected for an infant.

## Highlights

• Mesothelial cyst (MC) is very uncommon in clinic, which could occur in peritoneal, retroperitoneal or even pleural cavity.
• Retroperitoneal MC around hepatoduodenal ligament for an infant has never been reported before.
• We hereby described an infant patient with retroperitoneal MC who was misdiagnosed as congenital choledochal cyst.
• A local resection for the whole lesion under the da Vinci surgical system was unexpectedly performed during the operation.

## 1 Introduction

Mesothelial cyst (MC) is characterized as a large amount of mesothelial cells lined with the cyst wall, which is very uncommon in clinic, especially for pediatric patients [[1], [2], [3], [4]]. MC in small size is usually asymptomatic and sometimes detected by imaging examinations [5]. MC often manifests a well-boundary, watery-density and cystic lesion with no enhancement, which is often misdiagnosed as lymphangioma or pancreatic pseudocysts [[3], [4], [5], [6]]. We described an infant patient with retroperitoneal MC who was misdiagnosed as congenital choledochal cyst (CCC) and finally resected under the da Vinci surgical system, which has not been mentioned before in the literature. We stated that our work has been reported in line with the SCARE criteria [7].

## 2 Case presentation

**Fig. 1:**
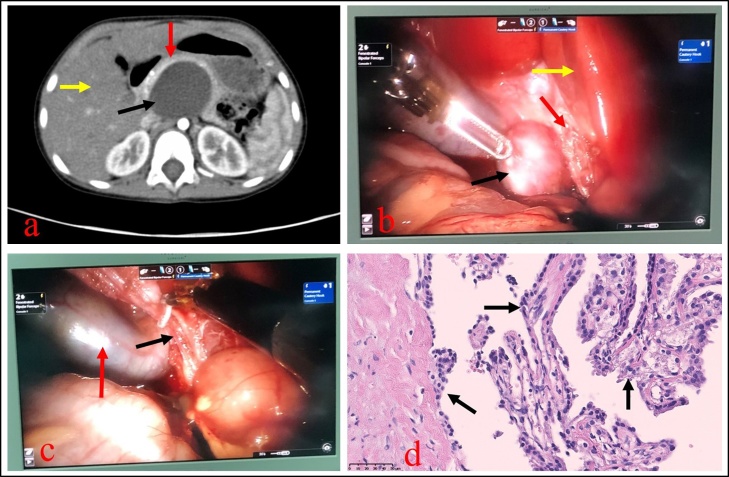
**a:** The preoperative abdominal contrast-enhanced computed tomography examination revealed a well-defined cystic mass in the hepatoduodenal ligament (black arrow), the pancreas was pushed forward (red arrow) and the intrahepatic bile duct was not expanded (yellow arrow). **b:** The dissociated cystic mass was intraoperatively found to be located behind the hepatoduodenal ligament (black arrow), the common bile duct was not dilated (red arrow), and the liver texture was normal (yellow arrow). **c:** The retroperitoneal cyst mass was totally resected from its pedicle under the minimally invasive da Vinci surgical system (black arrow), the gallbladder was also normal (red arrow). **d:** Hematoxylin and eosin staining of the pathological sections of the surgical specimens (magnification, ×400.) indicated that a large amount of mesenchymal cells was located in the cyst wall (black arrows).

A 2-year and 8-month girl infant was admitted into our hospital in November 2018, while discovering a gradually increscent epigastric cystic mass over 2 years, without any special treatment. This patient weighed 14 kg, with a height of 94 cm. This patient was asymptomatic, and her physical examinations showed no abnormity, such as fever, jaundice, abdominal tenderness and muscle tension. The liver function tests and the tumor markers from the patient’s blood were also within the normal range. The abdominal color Doppler ultrasonography and contrast-enhanced computed tomography both detected a 4 cm, round, cystic, and non-enhancing lesion in the hepatoduodenal ligament, without any obvious dilation of intrahepatic bile duct (Fig. 1a), in which CCC was still highly suspected by both radiologists and clinicians.

With her parents’ desperate determination for an operation, a da Vinci robot-assisted choledochal cyst resection with hepatojejunostomy was routinely prepared for this infant by excellent pediatric surgeons. During the operation, a well-defined retroperitoneal cystic mass arising from the posterior part of the hepatoduodenal ligament was detected, with grossly normal common bile duct, liver and pancreas (Fig. 1b). The retroperitoneal cyst mass was totally dissociated and excised using electrocautery under the minimally invasive da Vinci surgical system (Fig.1c). This patient recovered well postoperatively. The abdominal plasma drainage tube placed intraoperatively at the foramen of the omentum was pulled out on the fourth day after the operation. This infant was discharged from our hospital on the sixth postoperative day without any specific complication. The surgical specimens were histopathologically diagnosed as a retroperitoneal MC (Fig. 1d).

## 3 Discussion

MC is clinically very uncommon, especially for pediatric patients, which could occasionally occur in peritoneal, retroperitoneal or even pleural cavity [[1], [2], [3], [4]]. MC in small size is usually asymptomatic and sometimes detected by imaging examinations or intraoperative explorations. When a MC increases in size, nonspecific symptoms due to the compressive effect of the cyst on surrounding structures, such as abdominal or lumbosacral pain, constipation, jaundice and vomiting may develop [5]. Imaging examinations for MC often detects a well-boundary, watery-density and cystic lesion with no enhancement [6]. Due to the rare morbidity, as well as lacking of typical symptoms or signs and enough understanding by clinicians for this disease, MC is easily misdiagnosed as other illnesses, such as lymphangioma, pancreatic pseudocysts and tumor [[3], [4], [5]].

MC for pediatric patients could occur in the diaphragm [6,8] and liver [9] in the literature, while retroperitoneal MC around hepatoduodenal ligament for an infant has never been reported before, which might be easily misdiagnosed as CCC by radiologists or clinicians and routinely prepared a choledochal cyst resection with hepatojejunostomy. With a benign biological behavior, MC should be excised thoroughly to prevent recurrence, which could be performed by minimally invasive surgery, such as laparoscopic technique [3,6]. As the most advanced surgical method, da Vinci surgical system has been widely applied in adult gastrointestinal, hepatopancreatobiliary, urological and gynecological surgery in the past 10 years, which could provide more accurate operation for surgeon and less trauma for patient [10]. This safe and effective technique has also been recently performed for congenital malformations such as CCC and abdominal solid tumors such as nephroblastoma in pediatric surgery [11]. In the present case, although a CCC was highly suspected and a routine choledochal cyst resection with hepatojejunostomy was prepared preoperatively, the unexpected intraoperative findings of a retroperitoneal MC near the hepatoduodenal ligament didn’t affect her operation. On the contrary, with the help of clear surgical visual fields and elaborate surgical operations by da Vinci surgical system, the retroperitoneal MC was successfully resected for this infant. A local resection for the whole lesion would avoid the damages of surrounding tissues, as well as the recurrence of MC which still required a close follow-up afterwards.

## Declaration of Competing Interest

We declared that we had no conflict of interest among the authors.

## Funding

This research did not receive any specific grant from funding agencies in the public, commercial, or not-for-profit sectors.

## Ethical approval

Our research was approved by the Institutional Review Board of West China Hospital, Sichuan University.

## Consent

Written informed consent was obtained on admission from the patient’s parent.

## Author contribution

In this paper, M. Yang contributed to this work as first author; B. Xiang contributed as senior author. M. Yang extracted the data and wrote the manuscript. X.L. Xie made the figures. K.W Li made the references review. F.Y. Li and B. Xiang had important intelligent contributions and critically revised the manuscript. All authors read and approved the final manuscript.

## Registration of research studies

This is a retrospective case report, in which Registration of Research Studies seems to be unnecessary.

## Guarantor

B. Xiang is the corresponding author who accept full responsibility for the work.

## Provenance and peer review

Not commissioned, externally peer-reviewed.
